# Open questions in origin of life: experimental studies on the origin of nucleic acids and proteins with specific and functional sequences by a chemical synthetic biology approach

**DOI:** 10.5936/csbj.201402004

**Published:** 2014-02-23

**Authors:** Katarzyna Adamala, Fabrizio Anella, Rafal Wieczorek, Pasquale Stano, Cristiano Chiarabelli, Pier Luigi Luisi

**Affiliations:** a Sciences Department, University of Roma Tre; Viale Guglielmo Marconi 446, 00146 Rome, Italy; b MIT Media Lab, Departments of Brain and Cognitive Sciences and Biological Engineering, Massachusetts Institute of Technology, 77 Massachusetts Avenue, Cambridge, Massachusetts 02139, USA; c Department of Bionanoscience, Kavli Institute of Nanoscience Delft University of Technology Lorentzweg 1, 2628 CJ, Delft, The Netherlands; d FLinT Center, Institut for Fysik, Kemi og Farmaci (IFKF), University of Southern Denmark; Campusvej 55, 5230 Odense, Denmark; # These Authors are listed in alphabetic order and contributed equally to the work

**Keywords:** fragment condensation, organocatalysis, origin of life, random proteins, random RNA, sequence space, Ser-His

## Abstract

In this mini-review we present some experimental approaches to the important issue in the origin of life, namely the origin of nucleic acids and proteins with specific and functional sequences. The formation of macromolecules on prebiotic Earth faces practical and conceptual difficulties. From the chemical viewpoint, macromolecules are formed by chemical pathways leading to the condensation of building blocks (amino acids, or nucleotides) in long-chain copolymers (proteins and nucleic acids, respectively). The second difficulty deals with a conceptual problem, namely with the emergence of specific sequences among a vast array of possible ones, the huge “sequence space”, leading to the question “why these macromolecules, and not the others?”

We have recently addressed these questions by using a chemical synthetic biology approach. In particular, we have tested the catalytic activity of small peptides, like Ser-His, with respect to peptide- and nucleotides-condensation, as a realistic model of primitive organocatalysis. We have also set up a strategy for exploring the sequence space of random proteins and RNAs (the so-called “never born biopolymer” project) with respect to the production of folded structures. Being still far from solved, the main aspects of these “open questions” are discussed here, by commenting on recent results obtained in our groups and by providing a unifying view on the problem and possible solutions. In particular, we propose a general scenario for macromolecule formation via fragment-condensation, as a scheme for the emergence of specific sequences based on molecular growth and selection.

## 1. Introduction

One of the main open questions in the field of the origin of life is the biogenesis of proteins and nucleic acids as ordered sequences of monomeric residues, possibly in many identical copies. The first important consideration is that functional proteins and nucleic acids are – chemically speaking – *copolymers*, i.e., polymer formed by several different monomeric units, ordered in a very specific way. There are some indications of how homo-oligo/polypeptides [1–3] and homo-oligo/polynucleotides [4–6] can be obtained. Experimental results generally focus on exploring the conditions for achieving the desired condensation reactions and demonstrating, as proof-of-concept, that oligo- and polymerization can occur from the chemical viewpoint.

Attempts to obtain copolymers, for instance by a random polymerization of monomer mixtures, yield a difficult to characterize mixture of all different products. To the best of our knowledge, there is no clear approach to the question of the prebiotic synthesis of macromolecules with an ordered sequence of residues. Note that this problem has been beautifully solved by the invention of genetic code and the translation apparatus; however this does not solve the original question about how primitive functional molecules have arisen under prebiotic conditions.

The copolymeric nature of proteins and nucleic acid challenges our understanding of origin of life also from a theoretical viewpoint. The number of all possible combinations of the building blocks (20 amino acids, 4 nucleotides) forming copolymers of even moderate length is ‘astronomically’ high, and the total number of possible combinations it is often referred as the “sequence space”. Simple numerical considerations suggest that the exhaustive exploration of the sequence spaces, both for proteins and nucleic acid, was physically not possible in the early Universe, both for lack of time and limited chemical material. Then, the question becomes: even taking for granted a prebiotic chemical route to copolymerization (i.e., a condensation reaction occurring in primitive conditions that does not produce only simple homopolymers), how could early, functional biological macromolecules have been selected? Do other functional biological macromolecules exist in still-unexplored regions of sequence space? What do they look like? Could living systems have been originated if chemical evolution was allowed to start from other regions of sequence space?

In order to answer these questions and shed some light in the mystery of origin of life, we started some years ago a multifaceted research program aimed at clarify some aspects of the problem, by following a chemical synthetic biology approach. As it will be discussed below, we have recently proposed that small organic molecules could have played a key role in favoring condensation reactions (organocatalysis), and that the stepwise condensation of short, easy-to-form oligomers can give rise to very specific long sequences, in high copy number, due to the interplay between reactivity, selection and solubility (“fragment condensation”). Finally, we have devised a strategy to explore the sequence space of proteins and nucleic acid based on random sequences that make use of modern molecular biology methods, at the aim of search for those sequences that are folded, because folding is the most important pre-requisite for any biochemical activity.

Although these approaches do not represent the solution, they may indicate a very useful path in the right direction.

## 2. Formation of peptide bonds and phosphodiester bonds by organocatalysis

Because of their place as the most fundamental biopolymers, prebiotic synthesis of peptides and nucleic acids has been an object of many investigations. The earliest experiments on the condensation of amino acids were conducted by simply heating the solutions of amino acids [7] and various thermal condensation and dehydration reactions between amino acids or amino acid precursors appeared in the literature [8, 9]. Subsequent, more sophisticated approaches included hydrolysis of HCN polymers [10], condensation on clay environments [11] and in the presence of different mineral surfaces [12], or by atmospheric gases [13, 14]. Modern methods, such as copper-catalyzed and salt-induced peptide synthesis [15, 16], or condensation of *N*-carboxyanhydrides [17] allow for formation of peptides that are longer (ca. 10-mer) and can be formed from various proteinogenic amino acids.

Analogously, the first attempts at prebiotic condensation of nucleic acids constituted of simple heating experiments [18], or usage of metal ions [19]. The most effective methods that have eventually emerged are montmorillonite condensation [20, 21] and water/ice eutectic approach [22, 23].

Although there is number of interesting approaches for prebiotic condensation of both peptides and nucleic acids, they all have some shortcomings; mainly in the formation of specific sequences, and in chemoselectivity and regioselectivity. This problem might be successfully attacked if we take similar approach as life did – by exploiting organocatalysis. Enzymes are basically highly evolved organocatalysts. The investigation of prebiotic analogues possessing organocatalytic properties can shed light on the origin of molecular recognition and reactivity in the synthesis of proteins and nucleic acids and also provide novel perspectives (to be tested in the laboratory) about the “co-evolution” of peptides and nucleotides. This is perhaps the most intriguing aspect of organocatalysis in origin of life scenarios.

### 2.1 Peptide bonds

**Figure 1:**
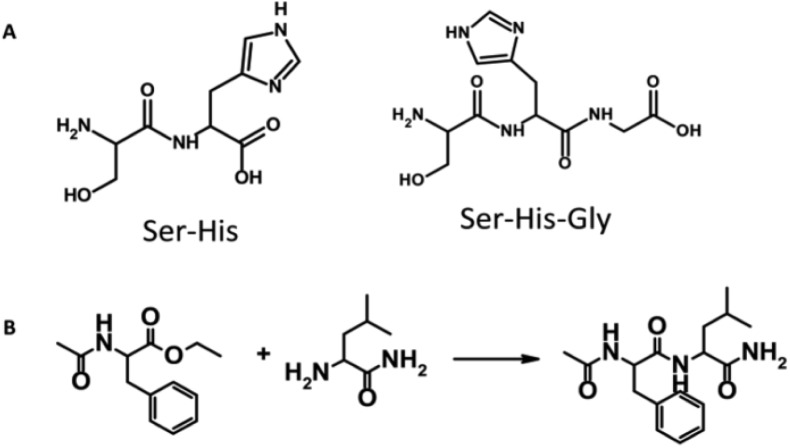
(A) The structure of catalytic dipeptides Seryl-Histidine (Ser-His) and Seryl-Histidine-Glycine (Ser-His-Gly). (B) The model reaction catalyzed by the dipeptides.

The simplest dipeptide Gly-Gly was reported to possess some proteolytic abilities [24–26] – but by far the best example of proteolytic activity is given by the dipeptide Ser-His [27]. Like Ser-His, also Ser-His-Gly is an active catalyst (see Figure 1A for structures), as well as similar di- and tri-peptides [27–29].

It is useful to remind that a catalyst does not change the free energy of reactants and products, but just enhance the reaction rate. If, by any means, the energetics of a reaction is inverted (i.e., by changing conditions so that the reaction goes in opposite direction), the same catalyst will still enhance the reverse reaction rate. Therefore, since Ser-His is endowed with proteolytic activity, under specific conditions it also acts as a catalyst for peptide *synthesis*.

Ser-His (and Ser-His-Gly) catalyzes peptide bond formation between a *C*-terminus of an activated amino acid and the free *N*-terminus of another amino acid. The activating group on the *C*-terminus is a carboxylic acid ester, most commonly in the form of methyl or ethyl ester.

Together with the condensation product, the main compound accumulating during the reaction is the product of hydrolysis of the ester of the activated amino acid. The yield of the hydrolysis product (the free acid), under most typically explored conditions, pH 7-8, in water-organic solvent mixture, accounts for half or more of the observed total substrate conversion. Thus the accumulation of the desired peptide is hampered by the hydrolysis [28].

The most extensively studied reaction catalyzed by Ser-His is condensation between two natural amino acid substrates: a *N*-acetylated phenylalanine ethyl ester and leucine amide (see Figure 1B), to give the hydrophobic dipeptide *N*-acetylated phenylalanyl-leucinamide [28]. The reaction has almost no uncatalyzed background product formation, and the product accumulation in presence of small peptide catalysts (most notably Ser-His or Ser-His-Gly) is easily detectable thanks to the high UV absorbance of the product and substrates. HPLC analysis is usually employed to monitor the progress of the reaction.

To date, the only known peptide condensation reactions efficiently catalyzed by small peptides are the ones with a great change in hydrophobicity between product, the byproducts and the substrates. For the reaction to proceed to the desired direction, the difference in solubility between substrates, the side product (the hydrolyzed activated amino acid) and the main product must be enough to remove the main product from the reaction mixture. Otherwise the only compound accumulated with significant quantity will be the hydrolysis product from the ester activated amino acid substrate.

The removal of the produced peptide from the reaction mixture can happen through the precipitation or through the migration of the product to a different phase. In the first case, the substrates must be fully soluble in the reaction mixture, and the product must be nearly completely insoluble [28]. In the second case, the reaction must be carried out in a two-phase system, for example with a lipid bilayer membrane as a hydrophobic environment. The product of the reaction accumulates in the bilayer membrane, thus being effectively removed from the reaction environment, shifting the equilibrium toward the formation of the dipeptide product [30].

Our groups have tested several model reaction and conditions to find a reaction catalyzed by small peptide that would not require removal of products to drive the reaction toward condensation [28–30]. To our knowledge, saturating solution with one or both of the substrates, changing activation groups (different esters on the activated amino acid), pH and other reaction conditions, did not result in formation of products soluble in the same phase as substrates and the catalyst [Wieczorek and Adamala, unpublished data].

It is conceivable that the small catalytic peptides could be obtained in an autocatalytic reaction. However, all efforts to create an autocatalytic cycle with Ser-His or other small proteolytic peptide have not been successful, most probably because of the above-mentioned requirement for the difference in solubility of the reactants.

The main limitation of the Ser-His catalysis (the requirement for insoluble product) could actually be advantageous in prebiotic selection scenario. It is possible to envision a selection driven by the insolubility of the products, especially in the presence of hydrophobic membranes. It could be possible that catalysis mediated by Ser-His (or similar small peptide) would allow for the synthesis of hydrophobic peptide chains beyond the lengths achievable by any other prebiotic chemistry synthesis.

The presence of the bilayer lipid membrane improves the yield of the reaction, minimizing the accumulation of the activated amino acid hydrolysis product. The accumulation of the main reaction product in the membrane is shown to cause changes in the stability of the bilayer membrane, and that leads to a mechanism of competitive growth of protocell liposomes containing the reaction product. Thus, the activity of small peptide catalyst has been coupled with the adaptive processes in the protocell vesicles [30].

It is possible that the efficiency of small catalytic peptides could be significantly improved by incorporating the Ser-His catalytic motif into a bigger oligopeptide. Addition of a substrate-binding domain, a residue(s) potentially stabilizing the catalyst-substrate transition state, could improve the ratio of the condensation product to the hydrolyzed substrate.

If the extensive hydrolysis of the activated substrate could be limited, the yields and efficiency of reactions catalyzed by Ser-His are expected to increase significantly. Potentially, more studies of the properties and limitations of the leaving group (the carboxylic acid ester) could yield activation/leaving group with lower hydrolysis rate, leading to increased yield of the desired peptide product.

### 2.2 Phosphodiester bonds

The formation of RNA chains can be catalyzed by the action of Ser-His dipeptide [29]. Under native, physiological-like conditions, Ser-His will hydrolyze nucleic acids [27]. The reverse reaction can be achieved only if we change the thermodynamic equilibrium [31]. By the introduction of water/ice eutectic phase we can lower the chemical activity of water, thus shifting the equilibrium towards condensation reactions [23]. To obtain such an environment, a reaction solution is cooled below its freezing point, but above the eutectic point. Under these conditions, most of the water is in the form of ice crystals and the other reactants are concentrated in the remaining liquid micro-inclusions, hence creating an environment with lower water activity in which condensation reactions can occur. This strategy has been used to achieve environmental conditions suitable for RNA formation.

**Figure 2:**
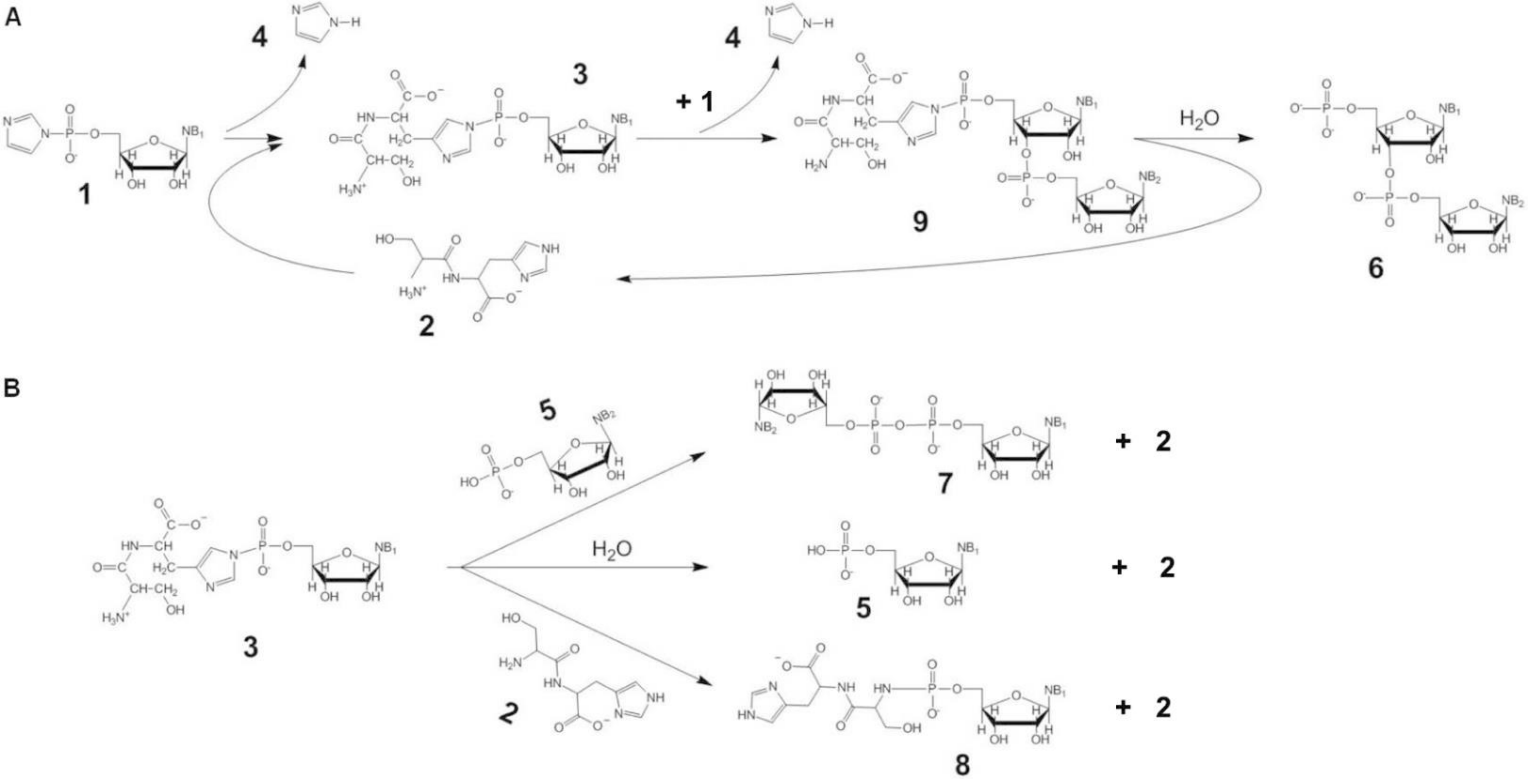
(A) The oligomerization of imidazole-activated nucleotides **1** by Ser-His **2** involves the creation of a covalent linkage between dipeptide and mononucleotide, **3**, releasing imidazole **4**. Compound **3** rapidly forms and is then slowly consumed. It can be attacked by 3′- or 2′-hydroxyl group from another nucleotide **1**, forming dimer **9** (imidazole **4** is released), which is hydrolyzed to give **6** (only 3’-5’ bonds shown, but the other possible attack from 2’-hydroxy group should also taken in account). (B) Schematic representation of the major side products. The intermediate **3** can produce also pyrophosphate dimer **7**, ribonucleotide **5** and a stable complex **8** are formed upon nucleophilic attack by a 5′-phosphate of **5**, water or the *N*-terminus of dipeptide Ser-His **2**, respectively (adapted from [29]).

To promote non-enzymatic RNA oligomerization the RNA monomers have to be activated by a good leaving group – usually an imidazole or its derivatives (Figure 2A, **1**).

**Figure 3:**
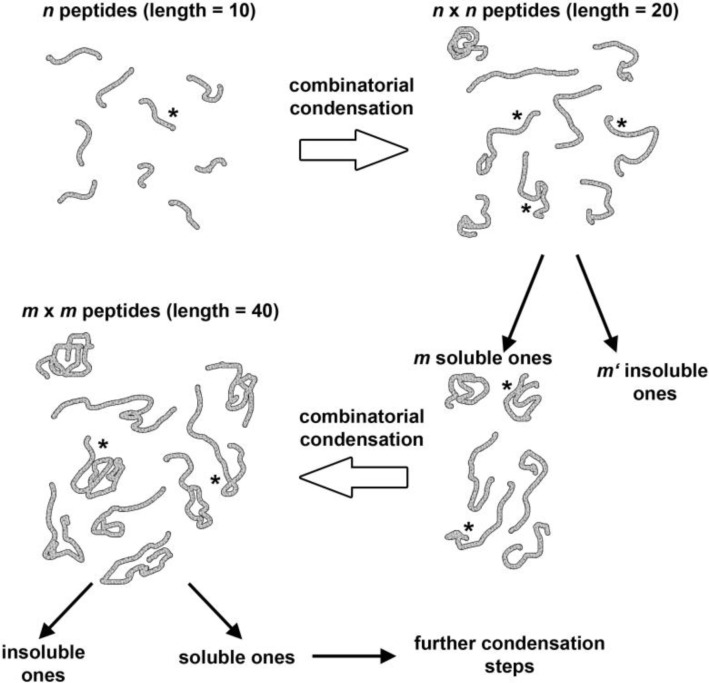
Condensation scheme of *n* peptides (10 amino acids long to yield ideally *n* ^2^ peptides (20 amino acids long), of which only *m* are soluble, and undergo further random fragment condensation yielding *m* ^2^peptides (40 amino acids long), of which only a few will be water soluble. By continuing this procedure, we reach long chains which are soluble and that can be seen as the product of a prebiotic molecular evolution. The asterisk indicates a catalytic center, capable of inducing peptide synthesis. The synthesis can be in principle also induced by an external catalytic peptide. Redrawn, with minor modifications, from [33].

The reaction proceeds through a transamination event, in which the histidine ring of the Ser-His dipeptide replaces the imidazole ring forming a short-lived peptide-nucleotide conjugate (Figure 2A, **3**). This reactive intermediate can then be nucleophilically attacked by other molecules of activated mononucleotide forming peptide-capped RNA oligomer (Figure 2A, **9**). Subsequently the peptide cap is hydrolyzed and we obtain unmodified single strand dinucleotide RNA fragment (Figure 2A, **6**).

The active intermediate complex **3** can be attacked by other nucleophiles present in the reaction's environment. Water will remain the most common nucleophile in the water/ice eutectic phase. Although its presence is drastically reduced comparing to liquid water environments, it is still high enough to hydrolyze not stable compounds like imidazole activated nucleotides **1** or peptide activated nucleotides **3**. Hydrolysis of those compounds results in the formation of unmodified nucleotide (Figure 2B, **5**). Unprotected phosphate group of **5** is a good nucleophile and its attack on activated mononucleotides results in the formation of pyrophosphate dimer (Figure 2B, **7**). Additionally, the amino group of the peptide can also attack activated mononucleotides resulting in the formation of inactive and long-lived peptide-nucleotide conjugate (Figure 2B, **8**).

Because of the competition of those side reactions the effectiveness of the prebiotic RNA synthesis it this setup does not exceed 10% [29].

Peptides are much more likely products of spontaneous condensation than nucleotide chains, therefore their potential as catalysts for the formation of RNA is very interesting from the origin-of-life perspective. The ability of simple peptides to catalyze RNA synthesis could represent a link between prebiotic chemistry and the RNA world, especially from a “co-evolutionary” viewpoint (i.e., the two most important classes of biological molecules that interacted from the very beginning with each other, thanks to molecular recognition, reciprocal stabilization, and auto- or reciprocal-catalysis).

The mechanism of Ser-His catalyzed RNA synthesis involves formation of an active intermediate which remains in equilibrium with the substrate. Such properties are characteristic of enzymatic catalysis. Enzymes are able to perform highly specific syntheses, thus creating many copies of the same sequence. We hope that simpler examples of organocatalysis like the catalytic dipeptide Ser-His can also exhibit some degrees of specificity. It is argued that various steric hindrances present in organocatalyst can influence the stereospecificity of reaction products. This premise is certainly true for enzymes, which are highly evolved examples of organocatalysis. However, the stereospecificity for Ser-His or other small peptides remains to be demonstrated. We expect the property of selective catalysis to be somewhat proportional to the length of catalytic biopolymer. Thus, enzymes - which are over 100 amino acids in length - do not have a problem obtaining high levels of stereospecificity. A catalytic dipeptide might not be long and complex enough to exhibit this property. However, we do believe that modification of simple peptide catalysts by addition of supplemental amino acid residues will eventually led to the emergence of stereospecific properties.

## 3. Chemical synthesis of *de novo* proteins

The previously discussed route to small oligopeptides or oligonucleotides certainly helps to understand the possible chemical origin of such molecules, and could also provide a rational explanation about the formation of copolymeric oligomers, which have the potentiality to further grow in size, fold, and become therefore functional (e.g., displaying catalytic activity). This problem is obviously more relevant for the case of proteins, because of the well-know sequence-structure-function interdependence.

As already mentioned, there are no methods described in the literature to efficiently generate long polypeptides, and we also lack a theory for explaining the origin of some macromolecular sequences instead of others. The method of the condensation of the *N*-carboxy anhydrides (NCA) as described by Commeyras and coworkers [2] offers perhaps a path to short polypeptides, around 10-15 residues in length, under alleged prebiotic conditions [3].

One idea to proceed further in the elongation, is to assume that certain short peptides (as for example Ser-His) or even some polynucleotides, or, more realistically, peptide-nucleotide complexes, can act as catalysts for the fragment condensation of the initially formed co-oligopeptides (it is remarkable that peptide synthesis in modern cells is catalyzed by RNA-protein complexes: the ribosomes). The actual nature of such catalysts is not known, however there is an interesting work which demonstrates that, as a proof of principle, that idea may be viable [32].

Figure 3 shows the key aspects of the model. A number *n* of say, deca-peptides can undergo a random combinatorial condensation yielding (ideally) *n* ^2^ 20-residues long peptides, and introducing here the constraint to keep only the soluble ones, only *m* of these peptides are left, that by further random fragment condensation give *m* ^2^ 40-residues long peptides, of which only a few will be water soluble.

The strategy of fragment condensation shown in Figure 3 was tested by Chessari et al. [32], operating by the Merrifield method for practical reasons, but assuming a prebiotic pathway. Eight random decapeptides peptides, whose sequences were generated by computers, were divided in two groups and randomly combined with each other to give 16 different 20-mers, which were individually synthesized. The four more soluble 20-mers where next combined with four random 20-mers, whose sequences were generated by a computer. Two out of 16 of the synthesized 40-mers resulted to be soluble in 6 M guanidinium chloride; and they were made partially soluble in aqueous buffer by adding a short hydrophilic tag (DDEE on *N*-terminus). In this way, finally, one 44-long protein was obtained, which was folded, and partly helical, as shown by a circular dichroism study – a novel protein obtained by pure chemical means.

## 4. “Never born” biopolymers

In addition to the investigation on the specific chemical routes leading to the emergence of functional biopolymers in origin of life scenario, a second unsolved question focuses on the relation between the sequence and the function of biopolymers.

The “Never Born Biopolymers” project aims at addressing the question of the design, synthesis and analysis of completely *de novo* biopolymers (proteins and RNAs), by exploring the sequence space for novel biochemical structures that do not exist in nature. These novel structures, in addition to their role in elucidating whether biologically functional biopolymers could have emerged from different “regions” of the sequence space (in origin of life scenarios), can be used as novel functional scaffolds for synthetic biology applications [34, 35].

The theoretical starting point is the fact that the number of natural proteins on Earth, although apparently large, is only a tiny fraction of all the possible ones. Indeed, there are thought to be roughly 10^13^ proteins of all sizes in extant organisms. This number, however, is negligible when compared to the number of all theoretically possible different proteins [36]. The discrepancy between the actual collection of proteins and all possible ones stands clear if one considers that the number of all possible 50-residues peptides that can be synthesized with the standard 20 amino acids is 20^50^, namely 10^65^. Moreover, the number of theoretically possible proteins increases with length, so that the related sequence space is beyond contemplation; in fact, if we take into account the living organisms, where the average length of proteins is much greater, the number of possible different proteins becomes even bigger [37]. The difference between the number of possible proteins (i.e. the *sequence space*) and the number of those actually present in living organisms is comparable, in a figurative way, to the difference that exists between a drop of water and an entire Ocean [33]. This means that there is an astronomically large number of proteins that have never been subjected to the long pathway of natural evolution on Earth: the ‘‘Never Born Proteins’’ (NBPs). Furthermore, the question whether a functionality is a common feature in the sequence space, or a rare result of natural selection, is of the utmost importance to elucidate the role of proteins in the origin of life and to fully exploit its biological potential and find new scaffolds for biological activities [33, 35].

In order to answer to this question, in a preliminary work we set up a method to test, as a first approximation, the folding stability of a random protein library. The protein library was generated starting from a totally random 150-nucleotides long DNA sequence library.

**Figure 4:**
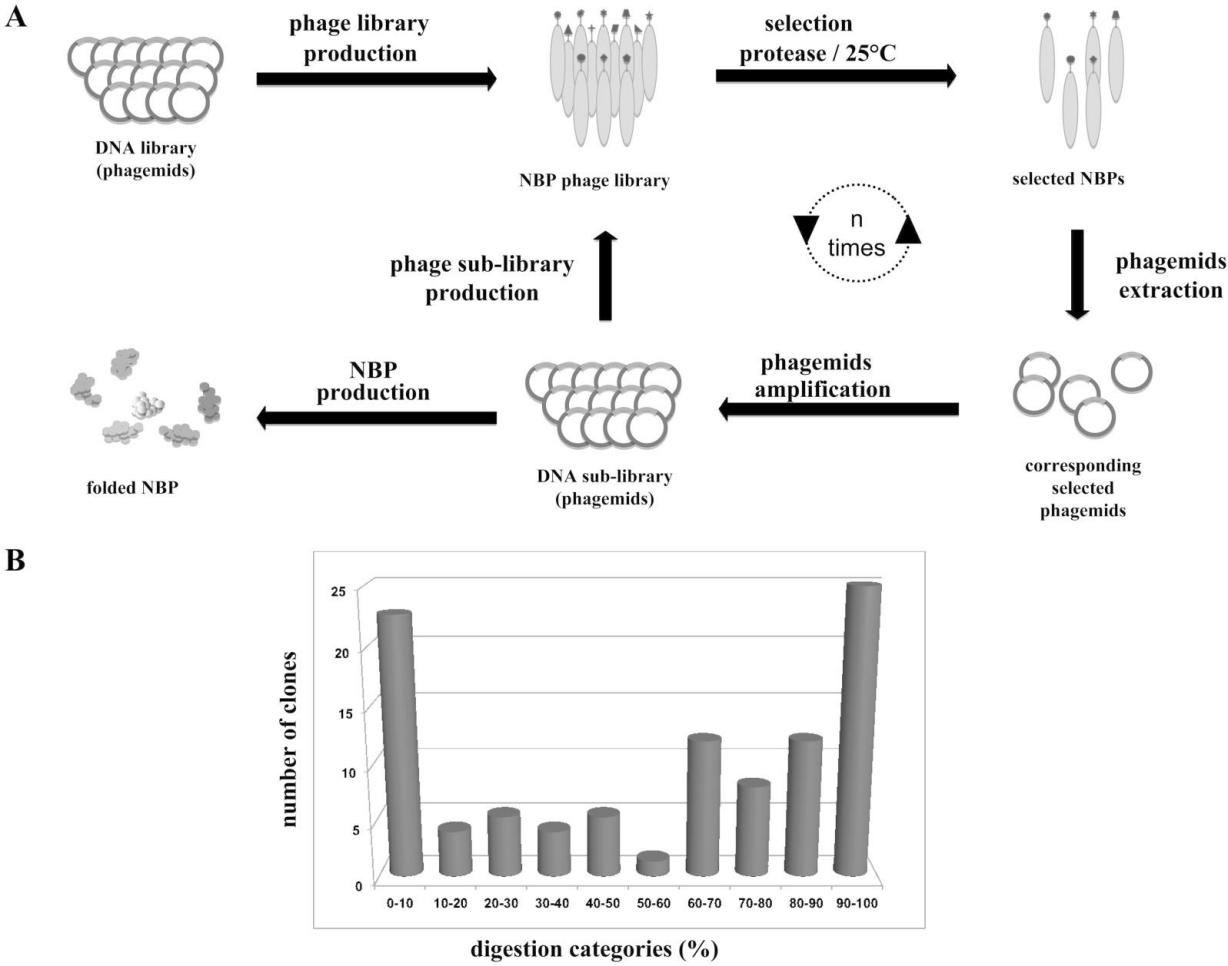
Never Born Protein. (A) Schematic representation of the NBP production and selection, using protease digestion to identify folded proteins (unfolded proteins are digested). The NBP fused with the bacteriophage coat protein in the phage library are selected at room temperature using a protease. The protein displayed corresponds to the genetic sequence within the phage. The corresponding selected phagemids are amplified and a new NBP sub-library of protease resistant proteins is created. The new phage library constitutes an enriched mixture, containing considerably fewer non-selected phage (i.e. non-resistant) than were present in the initial mixture. The cycle is optionally repeated one or more times, for further enriching the phage library in resistant proteins. (B) The tested NBPs are divided in categories (x-axis) according to the different levels of resistance against thrombin digestion shown by each sequence; y-axis shows the number of clones for each percentage of resistance [43].

The strategy was the introduction of a tripeptide sequence (-P-R-G-), which is a substrate for a protease, into the random sequence of polypeptide. It is expected that the proteins with a stable fold are much more resistant to proteolytic digestion than unfolded ones [38–42], so that they could be differentiated from all other non-folded ones during selection cycles (Figure 4A).

This work, based on phage display technique [33, 35, 43], allowed us to set up an efficient protocol for the production and study of NBPs and, in particular, the production of large libraries (on the order of 10^8^ – 10^9^ clones) of totally random 50 amino acid long proteins (note that 20 amino acids long peptides can be tested in similar way).

After the selection procedure, 75 of the resistant proteins (50 amino acids long) were analyzed individually by thrombin digestion assay (Figure 4B), and the best two sequences were chosen for a further structural analysis by circular dichroism. In native conditions, the two proteins were characterized by an alpha-helix plus beta-strands content of about 26 and 54%, respectively, indicating a significant folding, also confirmed by fluorescence analyses. Computer structural predictions, carried out by ROSETTA, predict rather compact 3D structures that agree with spectroscopic data. Moreover, 3D similarity search of the Protein Data Bank did not reveal any significant structural similarity between these two novel proteins and other proteins with known 3D structure [43]. This interesting result highlights the novelty of the fold predicted for these two proteins and can suggest a world of novel structured sequences with a great potential in terms of properties. Indeed, NBPs pose a series of challenging questions in basic and applied science regarding the possible novel principles of structure and catalytic properties that should be investigated in the future.

As described for random protein, the study of random RNAs poses similar considerations to take into account. The impressive difference between the number of possible proteins and the number of those actually present in living organisms [33] also hold for RNAs sequences. In fact, a single RNA sequence is only a point in a 4^*L*^ space of all the possible sequences of a given length *L* [33, 44]. This dimension 4^*L*^ comes from the fact that for a given polymer of length *L*, 4^*L*^ is the number of all the possible sequences, being each position of the sequence occupied by one of the four nucleobases.

Several independent investigations have shown that for a fixed-length polynucleotide, the number of possible sequences far exceeds the number of possible structures. Schuster and colleagues [44], using an inverse folding algorithm to calculate the number of different sequences with the same secondary structure, found that the sequence-space structures is the type of many-to-one and that the different sequences that take the same shape are spread evenly in the space of sequences, showing small or no degree of homology [45]. These theoretical indications are experimentally confirmed by the observation that only seven nucleotides are strictly conserved in the intron self-splicing group I [46], although the secondary structure of the ribozyme and its function are preserved. This case clearly illustrates the fact that sequences with little or no homology can achieve the same structure and perform the same function in spite of the diversity of their sequences.

The reasons behind the redundancy of the sequence/structure space of RNA can be found in the modular organization of the secondary structure of the RNA. Knight and colleagues have highlighted how RNA molecules are organized into independent functional domains connected by flexible spacers, whose sequences are irrelevant for the secondary and tertiary structures [47]. In particular, they developed a method to calculate the abundance of these functional domains, in a pool of random RNA sequences. The results showed that the modularity of RNA sequences further increases the redundancy of the sequence/structure space, since the number of ways that can be achieved a specific fold increases markedly due to the modularity of the domains of RNA.

In addition, the RNA secondary motifs, crucial for ribozyme activity, are generally due to the annealing of complementary bases that follow the Watson and Crick rules (A:U and C:G), increasing even more the probability of finding these domains in different regions of the sequence space and inside sequences not related to each other [48].

Consequently, RNA sequence space is rich in folded RNAs. In addition, the RNA sequence/structure space seems to be characterized by a high redundancy of related sequences that share the same structure, and probably the same function too.

On this basis we recently decided to explore the RNA sequence space in search of random RNA molecules that have a stable folding at high temperatures, by an assay set up in our lab: the RNA Foster (RNA Folding Stability Test). The RNA Foster assay determines the presence and thermal stability of secondary domains in RNA molecules by coupling enzymatic digestion with temperature denaturation. The assay gives the fraction of folded RNAs (*f*_fold_) as a function of the temperature [49].

**Figure 5:**
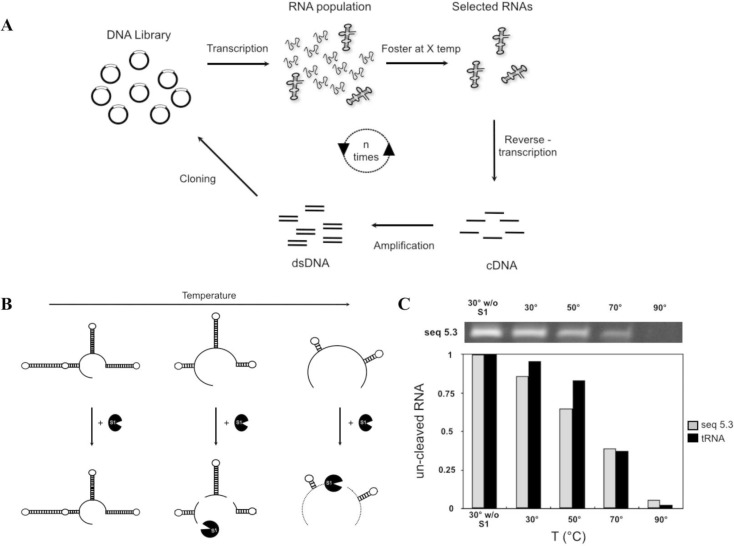
Never Born RNA. (A) Summary of the in vitro selection for folding ability. We transcribed the RNA population and we submitted it to a Foster incubation at 30 °C. After the test we recovered the un-cleaved RNA by reverse-transcription, using a RV primer and we produced the relative cDNA. At this point by PCR reaction we obtained a double-stranded DNA ready to be re-cloned in the vector. So the population can be transcribed again and a new cycle of selection can be started, increasing the temperature test and repeating this cycle. After each cycle of selection a representative number of clones of the population are recovered and sequenced. (B) Schematic representation of the RNA Foster Assay [49, 51, 52]. It combines the thermal incubation with the nuclease activity of nuclease S1. This assay allows evaluation of the folding ability of RNA. (C) RNA Foster on a single Never Born RNA sequence which presents a remarkable stability at 70 °C. Plot showing the residual random RNA amount (in gray) at the different temperatures. The fraction of folded RNA for each temperature is estimated by image analysis of the band intensity on PAGE urea gel (top of the figure), which corresponds to the amount of un-cleaved RNA after S1 incubation. The random sequence stability is compared with the one of the natural tRNA (in black).

The RNA Foster assay employs S1 nuclease, a single-strand-specific nuclease, to cleave single-stranded regions, thus monitoring the presence of double-stranded domains and indirectly any possible tertiary structure (Figure 5B).

Folded RNAs are more resistant to S1 nuclease than unfolded ones, as the latter are degraded faster than the former. The capability of nuclease S1 to work over a broad range of temperatures can be used to probe RNA secondary domain stability at different temperatures. An increase in temperature destabilizes the RNA fold, inducing either global or local unfolding. The fraction of folded RNA *f*_fold_ at each experimental temperature is determined by measuring the amount of RNA remaining after S1 digestion, using electrophoresis and a suitable staining method. The *f*_fold_ at different temperatures is used to assess the *T*_m_ of the RNA molecules. The Foster assay has several advantages: the simplicity and rapidity of execution, the requirement of simple instrumentation, and in particular the minimal RNA amount required. In general, the most notable advantage of this methodology is that it is a time-saving technique and is therefore suitable for the screening of large libraries and could be easily adapted for high-throughput studies, like in vitro selection experiments [50]. In particular, after exploring the folding rate for a library of 150 random bases [51], in the last years we have prepared and screened a library of totally random RNAs containing 60 residues (plus a pair of constant flanked regions for operational reasons). RNA Foster was successfully used to evaluate the structural properties of 18 random RNAs [52]. RNA molecules were tested individually at 4 different temperatures, in order to evaluate their ability to form internal double helices and, thus, to acquire secondary and tertiary structures. The results were used to assess the temperature of the main unfolding transition of individual RNAs. The experimental data show that almost all screened RNAs possess folded structures at 30 °C. Furthermore, half of the sampled RNAs maintain the fold up to 50 °C. Instead, at 70 °C all sequences, including the tRNA used as standard for the natural sequences, unfolded and were degraded by S1. Surprisingly, a sequence showed stability close to 50%, with a melting point around 70 °C and two random sequences were more stable than the tRNA at the same temperature (Figure 5C).

This data supports the assumption that the folding is not a unique property of biological RNAs (which underwent a lengthy process of evolution); by analogy this means that the sequence space of the Never Born RNAs could be full of folded stable structures.

Once again, it is worth noting that the tested RNA were chosen randomly and no selection was applied on them prior to analysis so thus finding a sequence stable approximately at 70 °C comes unexpectedly and supports the idea that thermostable RNAs may not be so rare in the RNA sequence space.

Despite the limited number of sequences explored by our technique, collected data suggest that the folding capability is a relatively common property, almost an intrinsic feature, of RNAs. Accordingly, RNAs have the capacity to fold into compact structures, even in the absence of selective pressure. In other words, the stable fold appears to be an intrinsic tendency, as already mentioned in our previous work for longer RNAs [51].

Since a well-folded structure is assumed to be, to large extent, a prerequisite for catalytic activity of RNA, and the planar secondary structure is a building block of the folding event, it is often justified to use secondary structure as a proxy for function. This one is particularly true for short sequences, since the probability of displaying tertiary contacts increases with the sequence length [53], leading us to claim that the possibility of a spontaneous emergence of a potentially functional RNA is a highly probable event.

This is an important issue not only for the origin of life research. In fact, from a more applicative viewpoint, typical of synthetic biology, the fact that folded RNAs are rather common in random RNA libraries means that the probability of identifying potentially functional RNAs *via* screening of large RNA pools becomes high.

In light of the results obtained from the studies on the stability of single Never Born RNAs by RNA Foster, we have started an extension of our work relying on in vitro selection [54], a technique based on the fundamental principles of Darwinian theory, which allows the exploration of functional properties of DNAs, RNAs and proteins in a quick, systematic and direct way. Our next goal is the selection of functional RNA from large random libraries. Beginning with a broad population of RNAs we applied a selective pressure, then we recovered, reverse-transcribed and amplified once again those molecules which satisfy the established condition. Then the population can be submitted to a new in vitro selection cycle. By doing this we isolated sequences that satisfy a well-determined request, in our case the ability to get a stable fold at the test temperature (Figure 5A). Preliminary results show that by using an in vitro selection approach is possible the isolation of sequences with a stable fold up to 80 °C degrees (unpublished data).

## 5. Concluding remarks

In this mini-review we have presented an unifying view of our chemical synthetic biology approach to understanding the chemical origin of primitive biomacromolecules and the exploration of the sequence space that characterizes them.

A possible chemical route to condense building blocks (or oligomer fragments) and create longer molecules has been shown. This is based on small molecule catalysis (organocatalysis). We have shown how Ser-His, a simple dipeptide catalyzes the condensation of activated amino acids or activated nucleotides, but other small molecules (perhaps short peptides that could be formed spontaneously) can also show similarly intriguing catalytic properties. Next, in order to provide a simple model for the generation of long sequences, in particular polypeptides, we have presented a realistic “fragment condensation” model, consisting in the combinatorial growth of short fragments, followed by selection of only some sequences for example for physical reasons (solubility). The effectiveness of this model has been demonstrated by a proof-of-concept experiment, where a soluble 44 amino acid long peptide has been obtained from random fragments.

Finally, we have introduced the project of “never born biopolymers”, aimed at selecting, by means of high-throughput screening among a large number of random sequence biopolymers, only those structures that satisfy certain criteria, like a stable fold, or a target catalytic activity. The interplay between structure and function plays a central role for the theoretical understanding of the sequence space.

We are convinced that by a chemical synthetic biology approach it is possible to study scientific problems that are difficult or impossible to approach in standard way (i.e., “taking apart” or analytical approach). The novel structures and strategies found by the synthetic biology approach, in addition of shedding light on origin of life questions, can also be relevant for developing biotechnological applications.
